# Evidence of a New Crystalline Phase of Prednisolone Obtained from the Study of the Hydration–Dehydration Mechanisms of the Sesquihydrate

**DOI:** 10.3390/pharmaceutics15061694

**Published:** 2023-06-09

**Authors:** Aurélien Lemercier, Nicolas Couvrat, Yohann Cartigny, Morgane Sanselme, Yohann Corvis, Philippe Espeau, Gérard Coquerel

**Affiliations:** 1 SMS, UR 3233, Université Rouen Normandie, 76000 Rouen, France; aurelien.lemercier@univ-rouen.fr (A.L.); nicolas.couvrat@univ-rouen.fr (N.C.); yohann.cartigny@univ-rouen.fr (Y.C.); morgane.sanselme@univ-rouen.fr (M.S.); gerard.coquerel@univ-rouen.fr (G.C.); 2 CNRS, INSERM, UTCBS, Chemical and Biological Technologies for Health Group, Université Paris Cité, 75006 Paris, France; yohann.corvis@u-paris.fr

**Keywords:** prednisolone, sesquihydrate, polymorphism, isomorphous dehydrate, hydration, dehydration, thermal analysis, X-ray diffraction, DVS, microscopy

## Abstract

The dehydration of prednisolone sesquihydrate is studied and characterized by different physico-chemical analysis methods. The meticulous study of this dehydration led to the highlighting of a new solid form (form 3), metastable, never identified before. In a second step, the rehydration of anhydrous forms 1 and 2 of prednisolone is studied, in particular by Dynamic Vapor Sorption. It is then demonstrated that neither of the two forms is sensitive to humidity. By means of solid-gas equilibria, the sesquihydrate can only be obtainable from the isomorphic anhydrous form. Finally, a classification of the sesquihydrate is made, taking into account, in particular, the activation energy determined during dehydration.

## 1. Introduction

The pharmaceutical development of an active molecule designed for oral dosage requires the knowledge of its different solid states, which can be amorphous, co-crystals, anhydrous crystalline, or solvated crystalline [1]. In these last three cases, screening remains essential to know whether or not polymorphs exist [2].

For crystalline forms, whether solvated or not, the relative stability must be established to choose the most suitable form for developing a drug in order to guarantee its chemical and physical stability while taking into account the solubility, the rate of dissolution and, therefore, the related bioavailability.

Prednisolone ((8S,9S,10R,11S,13S,14S,17R)-11,17-dihydroxy-17-(2-hydroxyacetyl)-10,13-dimethyl-7,8,9,11,12,14,15,16-octahydro-6H-cyclopenta[a]phenanthren-3-one, Figure S1, is a worldwide used corticosteroid to treat types of allergies, inflammatory conditions, autoimmune disorders and even cancers when co-administrated with chemotherapies. It is chemically derived from cortisone. It exhibits a solid-state landscape with several crystalline phases; to date: two polymorphs (Form I and II; REFCODES: JIWPEL01 and JIWPEL respectively), and a sesquihydrate (1.5 H_2_O REFCODE: JIWPIP).

Prednisolone Form I crystallizes in the monoclinic P2_1_ space group, whereas Form II and the hydrate both crystallize in the orthorhombic P2_1_2_1_2_1_ space group [3]. From thermal dehydration of the sesquihydrate, the anhydrous prednisolone is reported to crystallize in Form I prior to melting [3,4]. However, this compound is thermally unstable and degrades upon melting when thermal analyses are carried out at low scan rates. Recent studies carried out by Differential Scanning Calorimetry (DSC) at scan rates up to 60 °C/min clearly show that the thermal degradation has been shifted to higher temperatures after melting and then giving access to the “true” melting values of prednisolone polymorphs [5]. Based on these results, Form I melts at 256.7 ± 0.9 °C with a melting enthalpy of 39.9 ± 0.3 kJ mol^−1^, while Form II melts at 249.3 ± 0.8 °C with a melting enthalpy of 42.6 ± 0.5 kJ mol^−1^ [5]. These results unambiguously established the enantiotropic relationship between both solid forms in accordance with the observations of former studies [3]. This conclusion was supported by experimental observation of the solid-solid transition at approximately 154 °C close to the calculated value obtained from the experimental melting data, equal to 158 °C [5]. However, the conversion was only partial and randomly observed.

If the polymorph energetic ranking of prednisolone seems to reach a scientific consensus, the thermal stability of its hydrated form is still under debate. The thermal behavior of the sesquihydrate has been primarily studied by Veiga and coworkers [4]. In this study, apart from a different nomenclature of the solid phases, the authors detail the dehydration of the hydrate by Thermogravimetric Analyses coupled with DSC (TGA-DSC) performed at 2 °C/min, i.e., at a low heating rate, with the first large endotherm attributed to dehydration. However, TGA mass loss was completed at 100 °C, whereas the peak of the endotherm was recorded between 120 °C and 130 °C. Above this complex phenomenon, an exothermic peak (recrystallization) took place at ca. 145 °C and has been proven to correspond to the recrystallization of Form I.

The thermal behavior of the hydrated phase has also been investigated by Suitchmezian [3]. Starting from the sesquihydrate, obtained from an acetonitrile/water 90:10 (*v*/*v*) mixture, a DSC run was performed at 4 °C/min, i.e., at a low heating rate, thus favoring degradation on melting. A highly energetic endothermic peak assigned to dehydration was revealed at around 99 °C corresponding to the maximum deflection of the peak, and then melting/degradation of the anhydrous Form I with a peak temperature equal to 245 °C and an “onset” melting point that can be estimated at approximately 220 °C. This was confirmed by the time-resolved X-ray powder diffraction (TR-XRPD) experiments performed, highlighting that the thermal dehydration of sesquihydrate directly leads to the anhydrous Form I. The behavior was thus slightly different from that observed by Veiga and coworkers [4] since no recrystallization peak after dehydration was observed by Suitchmezian and coworkers [3]. However, during our attempts to isolate prednisolone sesquihydrate in order to thoroughly study its dehydration process, such behavior was never encountered, and the dehydration mechanism appeared much more complicated than that reported by Suitchmezian et al. [3]. Moreover, a new solid phase has been evidenced during the dehydration process of prednisolone sesquihydrate.

In this paper, we present the detailed thermal behavior of prednisolone sesquihydrate as well as the influence of the relative humidity (RH) regarding the behavior of the two anhydrous solid phases and the sesquihydrate. Then, a classification from an energetic and mechanistic point of view is proposed.

## 2. Materials and Methods

### 2.1. Materials

Prednisolone Form I was supplied by Sigma Aldrich (St. Louis, MO, USA) with a purity grade >99%. Prednisolone Form II was supplied by Thermo Fisher with a purity grade >99%.

Due to the poor solubility of prednisolone in pure water, sesquihydrate crystallization was achieved in mixtures of acetone/water (90/10 *v*/*v*) either by the simple stirring of a suspension during 24 h (whatever the starting form in suspension) or by total recrystallization followed by partial evaporation of a homogeneous liquid.

### 2.2. TGA-DSC-MS

Thermogravimetry–Differential Scanning Calorimetry (TGA-DSC) analyses were performed on a TGA-DSC STA449C Netzsch. Dry samples of ca. 10 mg were weighted in 25 µL aluminum pans equipped with pierced lids. A heating rate of 1 °C/min up to 10 °C/min was applied between 20 and 260 °C under a helium atmosphere. Data treatment was performed by using Netzsch Proteus^®^ software v6.1. The chemical nature of escaping gases during heating was identified by using a Netzsch QMS 403 C mass spectrometer coupled with the 449C TGA/DSC apparatus.

### 2.3. XRPD and Temperature Resolved XRPD

XRPD analyses at room temperature were performed using a D8 Discover diffractometer (Bruker AXS, Billerica, MA, USA). The instrument is equipped with an X-ray tube containing a copper anticathode (λ = 1.54060 Å, 40 kV, 40 mA) and a kβ filter (Ni). The scan step was fixed at ∼0.04° with a counting time of 0.5 s/step over an angular range of 3–30°. Data were processed by using DIFFRAC.EVA software release 2018 (version 4.3.0.1).

Temperature-Resolved X-ray diffraction data were collected using a D8 series II diffractometer (Bruker AXS). The instrument is equipped with an X-ray tube containing a copper anticathode (40 kV, 40 mA) and a kβ filter (Ni). Temperature-Resolved XRD analyses were performed on the same apparatus equipped with a TTK 450 heating stage (Anton Paar, Graz, Austria). Every XRD scan was carried out with a scan step fixed at ∼0.04° and a counting time of 0.5 s/step over an angular range of 4–30°. Samples were heated at a rate of 2 °C/min between the stages and remained circa 5.5 min at every desired temperature.

### 2.4. Thermal Microscopy

Optical microscopy observations were performed with a Nikon Eclipse LV 1000 equipped with a Leica Flexacam C3 camera. Heating of the sample was performed under an inert atmosphere (Nitrogen) with a Linkam THMS 600 hot-stage cell at a rate of 5 °C/min. Data treatment of the pictures was performed with the Leica Application Suite X software version V5.02.24429.

### 2.5. Dynamic Vapor Sorption

Water sorption/desorption isotherms were carried out at 25 °C with a Dynamic Vapor Sorption apparatus (DVS advantage, Surface Measurement Systems, Wembley, UK). In this apparatus, the temperature (±0.5 °C) and the relative humidity (±0.1% RH) are regulated; the mass variation is recorded by a microbalance (±0.1 µg). Samples were submitted to the successive sorption–desorption cycles (between 0% and 90% RH per step of 10% RH). The fixed criterion for the step change was a mass variation of the sample lower than 0.0005% per minute in the limit of 2000 min per step.

### 2.6. Molecular Modeling

Molecular modeling calculations were performed using the Material Studio 2019 software (19.1.0.2353) [6]. Geometry optimizations were carried out with the forcite module using the COMPASS II forcefield [7,8] and the smart algorithm. Charges were implemented using the forcefield, and non-bonding interactions were computed using the Ewald option [9].

The method was based on the work of De Saint Jores et al. [10]. A unique cell was taken into account, geometry optimization was performed, and the energy of the packing was calculated (E_reference_).

## 3. Results and Discussion

### 3.1. Thermal Behavior of Prednisolone Sesquihydrate during Dehydration

**Figure 1:**
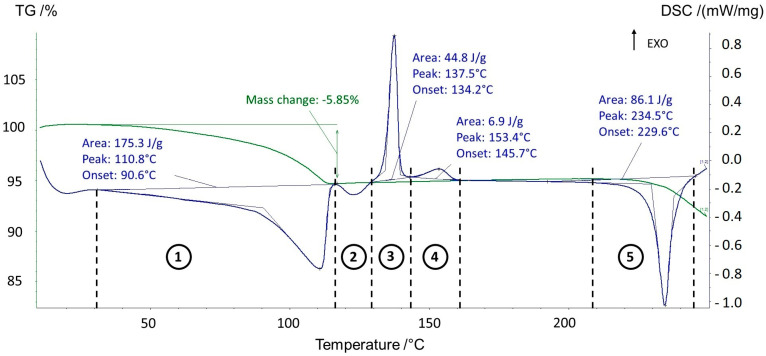
TGA-DSC analysis (5 °C/min) of prednisolone sesquihydrate. The green curve displays the TGA signal, whereas the blue curve displays the DSC signal.

Upon heating, the behavior of the sesquihydrate was significantly different from that previously reported [3]. Figure 1 displays the TGA-DSC of prednisolone sesquihydrate.

If the starting material (sesquihydrate) and the final one (Form I) were identified by X-ray powder diffraction, the pathway between the two states appeared unclear and thus necessitated further investigations.

**Figure 2:**
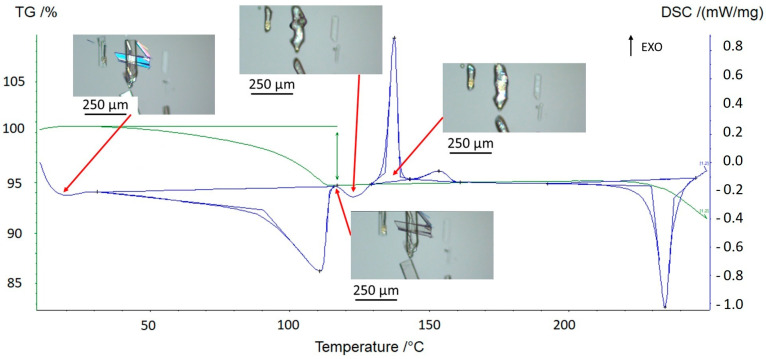
Thermal behavior of the sesquihydrate at 5 °C/min followed by hot-stage microscopy.

Hot-stage microscopy experiments (Figure 2) were carried out on prednisolone sesquihydrate in order to reveal the nature of the successive phenomena observed with TGA-DSC analyses.

**Figure 3:**
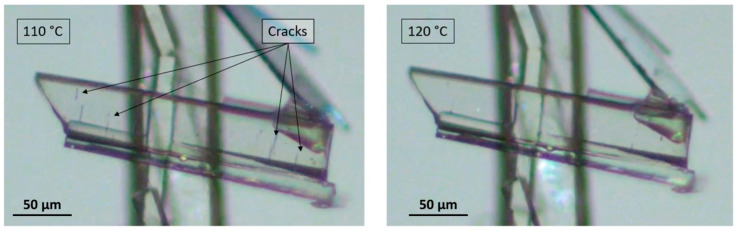
Microscopy pictures of a crystal of dehydrated prednisolone (Form III) at 110 °C exhibiting cracks (**left**) and the same crystal at 120 °C exhibiting healed cracks (**right**) just before the metastable fusion of the solid phase at 125 °C.

After dehydration (visually occurring at around 100 °C), the crystals display characteristic cracks perpendicular to the major growth axis of the crystal but keep their overall shape, edges, and facets [11]. On subsequent heating, these macroscopic defects disappeared, highlighting a structural analogy between the sesquihydrate and the corresponding dehydrated solid (Figure 3).

The crystal-to-crystal transformation clearly indicates that the resulting anhydrous form results from quasi-topotactic dehydration of the sesquihydrate (i.e., for the loss of water molecules without irreversible alteration of the crystal packing, only cracks appeared, which were subsequently healed) and could be characterized as an isomorphous dehydrate (i.e., an anhydrous form exhibiting a crystal packing similar to that of the sesquihydrate). This new anhydrous form will now be labeled prednisolone Form III.

**Figure 4:**
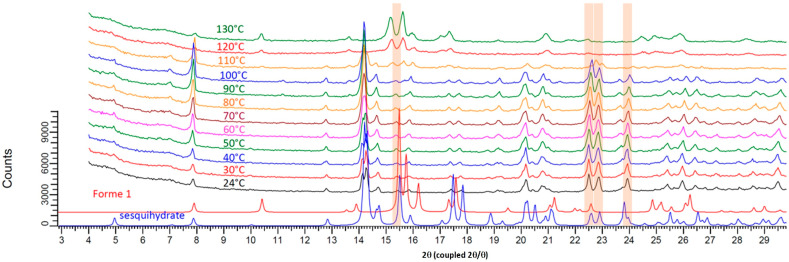
Temperature resolved XRPD patterns from 20 °C to 130 °C starting from prednisolone sesquihydrate raw material.

**Figure 5:**
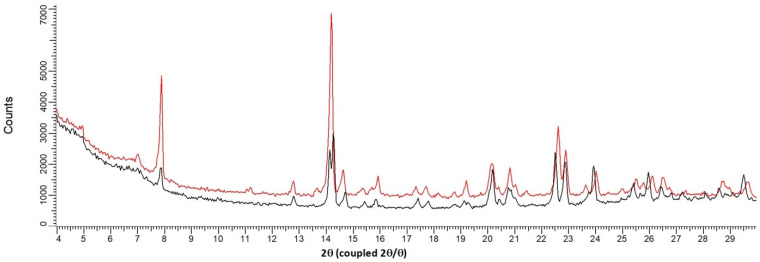
Overlay between XRPD patterns of the solid at 24 °C (sesquihydrate) and the solid at 100 °C (Form III: isomorphous dehydrate). Black curve: 24 °C, red curve: 100 °C.

Its structural filiation with the sesquihydrate was confirmed by TR-XRPD measurements (Figure 4 and Figure 5). Indeed, the pattern at 100 °C for the fully dehydrated sample is highly similar to that of the sesquihydrate, but specific peak shifts or peak merging could be noticed among them (14.3°, 22.6°, 22.9°, and 24°).

It should be noted that, in contrast to TGA-DSC experiments, TR-XRPD is not a fully dynamic experiment: the heating rate between every step scan (each 10 °C) was fixed at 1.8 °C/min, and the sample was kept at constant temperature during the 6 min XRPD scan. Therefore, the average heating rate of the analysis could be estimated to be lower than 1 °C/min. At this heating rate of 100 °C, the product was confirmed to be fully dehydrated (see Figure 1).

The reversible rehydration was proven by successive TGA-DSC analyses. After a first heating ramp at 5 °C/min up to 110 °C, the dehydrated sample was stored at room temperature for 12 h and, then a second heating ramp at the same scan rate was applied, confirming the full rehydration after storage under room temperature conditions.

**Figure 6:**
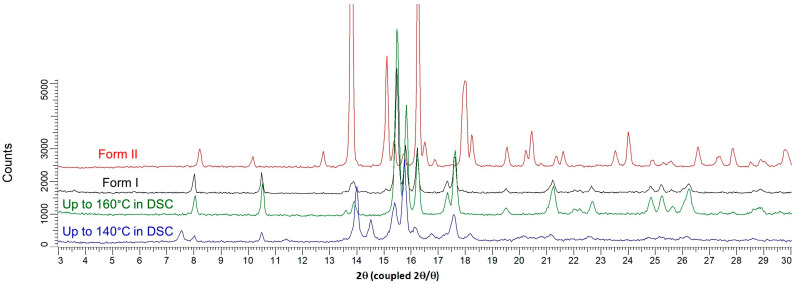
Comparison between XRPD patterns of the solid isolated after the first exothermic peak in DSC (blue curve), the solid isolated after the second exothermic peak (green curve), Form I and Form II of prednisolone (black and red curves, respectively).

From the hot-stage microscopy experiments (Figure 2 + three videos displaying heating from 100 °C up to 150 °C presented in Supplementary Materials), the small endotherm observed from DSC experiments at ca. 125 °C was assigned as the melting of the new solid Form III below the two other anhydrous forms. This melting process may partially overlap with the sharp recrystallization due to high overcooling (first exothermic peak at approximately 130 °C, zone 3 in Figure 1). The obtained solid was analyzed by X-ray diffraction and consisted of a mixture of defective Form I and II (Figure 6). The mixture of crystals then fully converts into Form I (second small exotherm at ca. 150 °C, zone 4 in Figure 1), which corresponds to the final solid phase of the sample (Figure 6).

The existence of an isomorphous dehydrate displaying a melting point far below the “usual” anhydrous forms has already been reported for several compounds by Stephenson [12] and presents great similarities with that of Rimonabant, which crystallizes (among several solvates) as a monohydrate whose “smooth” dehydration under dry atmosphere led to isolating an isomorphous dehydrate that melts more than 70 °C below the two anhydrous forms [11].

**Figure 7:**
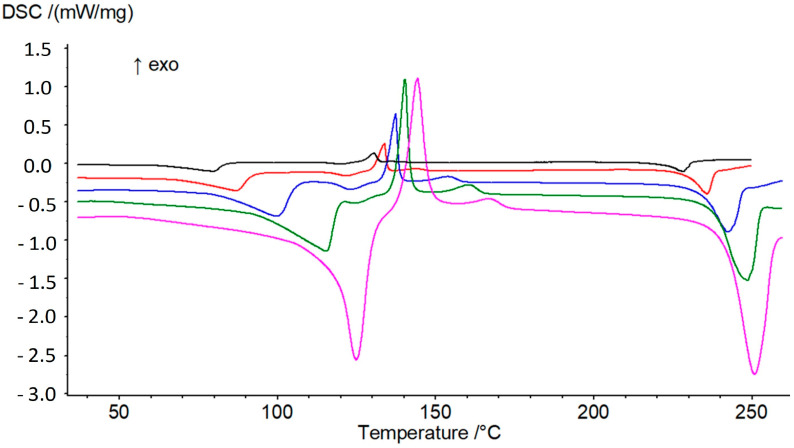
DSC analyses of prednisolone sesquihydrate at 1, 2, 5, 10 and 20 °C/min, from top to bottom.

The behavior of the sesquihydrate of prednisolone upon heating was studied at different heating rates from 1 to 10 °C/min by TGA-DSC analyses (Figure 7). As observed in Figure 7, the peak position of the dehydration process varies with the DSC heating rate. This indicates that the water loss can be treated from a kinetics point of view. In such a case, the transition was studied in non-isothermal conditions using the model developed by Kissinger [13].

Therefore, based on the displacement of the dehydration peak versus the heating rate, the activation energy can be estimated from the following equation [13,14]:

$$\ln\left(\frac{\varphi }{T_{p}^{2}}\right)=-\frac{E_{a}}{{RT}_{p}}+\ln\left[\frac{A\cdot R}{E_{a}}\right]$$

where φ is the heating rate, $T_{p}$ is the peak temperature, $E_{a}$ is the activation energy, A is the Arrhenius constant, and R is the ideal gas constant.

Consequently, the plot $\ln\left(\frac{\varphi }{T_{p}^{2}}\right)=f(\frac{1}{T_{p}})$ is a straight line with a slope equal to the opposite of the activation energy over the ideal gas constant. The linear regression performed on the experimental data led to an activation energy of about 68 kJ/mol with a linear regression correlation factor of 0.99 (see Figure S3, Supplementary Materials). If we refer to the literature, one can observe that the activation energy seems to increase with the dehydration temperature. Independently of the degree of hydration, for a dehydration temperature close to 90 °C, an activation energy value can be expected around one hundred kilojoules per mole of compound [15,16,17], somewhat higher than our value.

### 3.2. Hydration/Dehydration Behavior versus Relative Humidity of Prednisolone

**Figure 8:**
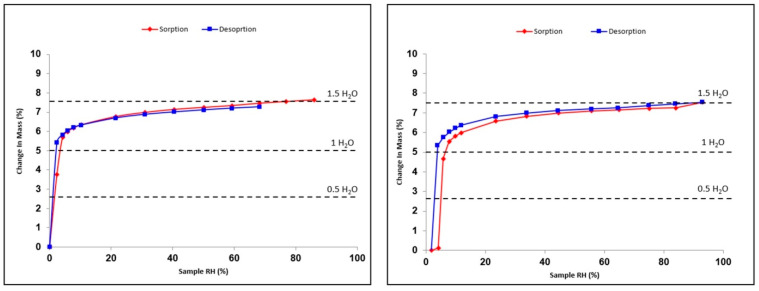
Sorption–desorption isotherms of prednisolone sesquihydrate at 24.9 °C (**left**) and 48.7 °C (**right**).

Both prednisolone solid phases I and II, as well as the sesquihydrate, were submitted to RH variations using DVS analyses at two fixed temperatures, i.e., 24.9 and 48.7 °C. From these experiments, it appears that both Form I and Form II of prednisolone are non-sensitive to humidity at room temperature and do not tend to uptake water even during long exposure to high RH. This non-hygroscopicity property of prednisolone has also been confirmed with supplementary experiments consisting of dispersing prednisolone powder in water followed by the evaporation of the latter. Furthermore, the sesquihydrate was also submitted to RH variations cycles (between 0% and 90% RH) at the same temperatures as the anhydrous forms (Figure 8).

First, we can conclude that the hydration–dehydration mechanism is perfectly reversible in the 0–2% RH domain) at 24.9 °C. This revealed the great stability of the sesquihydrate versus relative humidity, the latter being able to fully dehydrate only in extremely dry atmospheres. Moreover, the resulting anhydrous form isolated at 0% RH is highly sensitive to humidity as it fully rehydrates as soon as the RH increases. The anhydrous form obtained by soft dehydration at 24.9 °C is very likely to be different from Form I or Form II of prednisolone, where no hydration was observed, whatever the RH. This behavior is characteristic of an isomorphous dehydrate or channel hydrate [12,18], which could be considered a new anhydrous polymorphic form. Thus, we propose that Form III may be obtained from the smooth dehydration of the sesquihydrate at 0% RH. One can note that even with the increase of the temperature to 48.7 °C, the isomorphous dehydrate rehydrates between 2 and 4% RH.

The stoichiometry of the hydrate varies between 1 and 1.5, equivalent to the water molecule, depending on the RH value. This indicates that a continuum in water molecules content exists between 1 and 1.5 H_2_O, depending on the RH surrounding the solid (between 4 and 95% RH). Under dry conditions (i.e., between 0 and 2% RH), the equivalent of one water molecule is “extracted” from the structure without destroying the global organization of the periodic bond chain to obtain Form III. All of these experimental facts seem to indicate that the water molecules do not play the same role in crystal packing.

**Figure 9:**
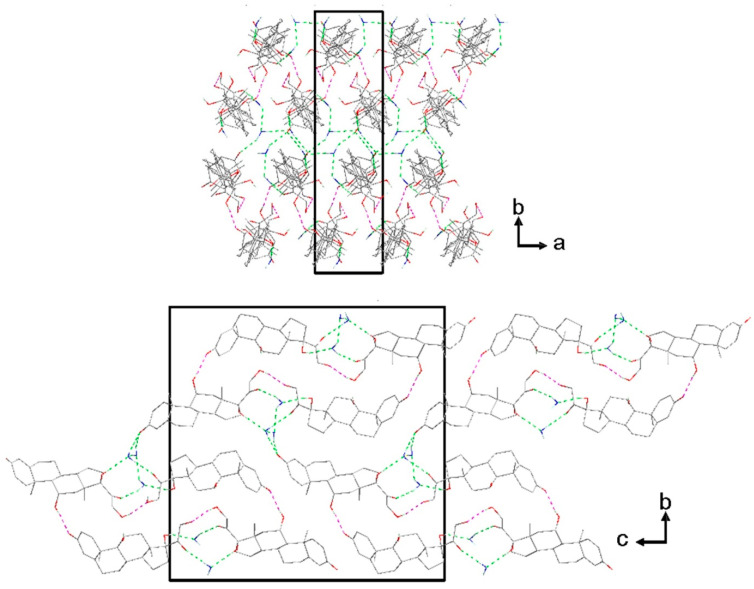
Crystal packing of prednisolone sesquihydrate along the c-axis (**up**) and the a-axis (**bottom**) (hydrogen bonds are displayed in dashed lines green or pink regarding if water molecules are involved or not, respectively).

In hindsight, from the DVS data, there are two regimens of water loss; thus, the activation energy calculated above is related to global activation. Figure 9 shows the crystal packing of prednisolone sesquihydrate.

**Figure 10:**
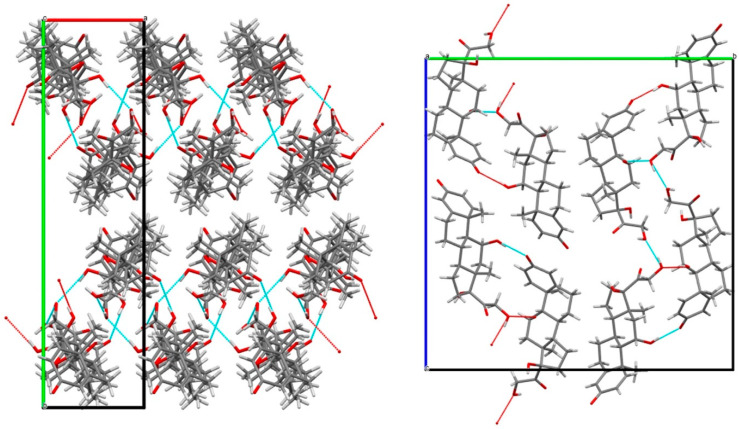
Crystal packing of prednisolone sesquihydrate (water molecules not shown) along the c-axis (**left**) and a-axis (**right**).

The cohesion of the crystal packing is ensured by both intermolecular H-bonds between prednisolone molecules and H-bonds between the water and prednisolone molecules, giving rise to a three-dimensional PBC network (see Figures S4–S9). An overlay between the two molecules of prednisolone present in the asymmetric unit of this sesquihydrate compound confirms their high similarity in terms of conformations (Figure S10). Even if the crystal structure of Form III has not yet been resolved yet, the crystal packing of prednisolone sesquihydrate has been considered without the water molecules (deleted from the crystallographic data of prednisolone sesquihydrate) and is presented in Figure 10.

**Figure 11:**
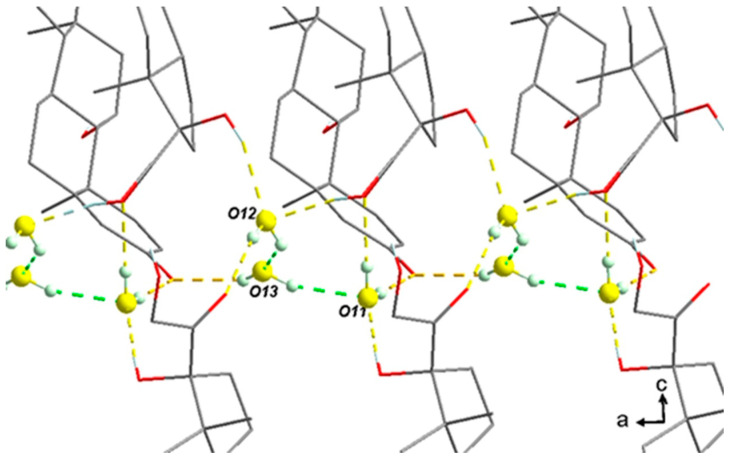
Cluster O12-O13-O11 of water molecules along the a-axis.

If water molecules are artificially removed from the crystal lattice, the cohesion of the structure can still be ensured by the intermolecular interactions (H-bonds and van der Waals interactions) between the prednisolone molecules, which is in accordance with the existence of an isomorphic desolvate and topotactic dehydration. Molecular modeling calculations performed on water departure of either one, two, or three water molecules from the packing did not reveal any clear energy difference between the removal of any water molecule (see Molecular Modeling part of Supplementary Materials). Nevertheless, the single-crystal data collection of the sesquihydrate was performed at 170 K, and the reliability index of the resolution reached 4.2%. At the end of the refinement, the isotropic thermal agitation (Beq) of the three water molecules were: U(O11) = 0.042, U(012) = 0.048 and U(O13) = 0.076. Two hypotheses can therefore be proposed:

(1) There is a slight compensation of some degrees of vacancy for O13 by a higher Beq. Consistently, several TGA analyses tend to converge to 1.3 equivalents of water molecules starting from a sample conditioned at room temperature and under an ambient atmosphere. This could therefore indicate the non-stoichiometric behavior of the sesquihydrate. The maximum stoichiometry (1.5 water molecules) is reached only with high RH (cf. DVS data).
(2) The three water molecules O12-O13-O11 form a cluster, as shown in Figure 11. The slight global deficiency in water molecules under ambient conditions should not be considered for a single molecule only but rather for the whole cluster. At room temperature for a RH higher than 2%, there is a strong trend toward approximately occupying 2/3 of the three positions. For higher RH, the last third is progressively filled.

By comparing the crystallographic structures of each solid form and in consistency with experimental data, it is possible to conclude that there is a destructive/reconstructive process between the sesquihydrate and Form I (or between sesquihydrate and Form II). Suitchmezian and coworkers have shown that even if the conformation of prednisolone remains the same in each solid form [3], there is no filiation between the three packings, which is in accordance with the observed behavior in the frame of the present study [19,20].

### 3.3. Classification of the Sesquihydrate

Several authors have proposed to classify the different hydrates as a function of different properties (e.g., the activation energy of dehydration or mechanistic classification (i.e., related to the nature of the product obtained after dehydration) [18,21,22,23]. Takahashi and Uekusa proposed a Dehydration–Rehydration Classification System (DRS) into 3 classes according to their hydration and dehydration activation energies [23].

With an activation energy of ca. 68 kJ/mol and a fully reversible hydration–rehydration mechanism, the transition between prednisolone sesquihydrate and Form III perfectly fits the average value for dehydration of Class 1 hydrates (hydrates displaying full dehydration–rehydration cycles) as ranked by Takahashi and Uekusa [23]. Due to the complexity of measuring rehydration energy, no data are provided for the rehydration activation energy.

Another model, Rouen-96, considers the mechanistic aspect of the dehydration and rehydration [18]. Based on this classification, the sesquihydrate falls into Class II, were a structural filiation between the mother and daughter phase is observed. However, access to the crystalline structure of Form III is required to choose between Class II-C.R. (structural filiation between mother phase and daughter phase with Cooperative Rearrangement) or II-T hydrate (structural filiation between mother phase and daughter phase without reorganization: Topotactic), depending on the possible relaxation of prednisolone molecules after the release of water. The healing of the crystals and the slight XRPD differences between sesquihydrate and Form III could indicate a relaxation in the crystal structure and a hydrate of Class II-C.R. according to the Rouen-96 model. In Galwey’s model [24] for dehydration, prednisolone sesquihydrate should be classified as WET-3 with a clear structural filiation between the sesquihydrate and its corresponding anhydrous phase. Nevertheless, this dehydration is associated with mechanical constraints that result in the formation of cracks.

## 4. Conclusions

This study proposes a complete version of the polymorphic landscape of prednisolone and, in particular, the behavior of the sesquihydrate upon dehydration. A new solid form of prednisolone has been isolated (i.e., Form III) and characterized as an isomorphous desolvate of the sesquihydrate. This daughter phase shows a reversible dehydration—rehydration phenomenon and extensive structure similarities with the corresponding mother phase due to a quasi-topotactic desolvation and resolvation mechanisms.

This study is a new case depicting the possibility of obtaining isomorphous desolvates through smooth dehydration. It also demonstrates that the exploration of a unary phase diagram can necessitate excursions in binary phase diagrams with solvents in order to discover new polymorphic forms thanks to the management of soft desolvations.

This new phase is, from a relative stability standpoint, the least stable of the three anhydrous prednisolone solid forms. From a pharmaceutical formulation perspective, such complex behavior poses real challenges, notably in raw materials and drug manufacturing. Indeed, its strong hygroscopic character makes storage difficult, and the rehydration seems so fast that it could jeopardize the benefit of a much higher solubility in an aqueous medium because rehydration could be faster than dissolution. A study of such competition needs to be assessed.

## Data Availability

No new data were created except those in this paper.

## Supplementary Materials

The following supporting information can be downloaded at https://www.mdpi.com/article/10.3390/pharmaceutics15061694/s1, Figure S1: Molecular structure of Prednisolone. Figure S2: Concomitant Mass Spectrum variation for *m*/*z* = 18 (red curve) during TGA-DSC analysis of Prednisolone sesquihydrate (5 °C/min). Figure S3: Kissinger plot curve: $\ln\left(\frac{\varphi }{T_{p}^{2}}\right)=f(\frac{1}{T_{p}})$ for dehydration peak of prednisolone sesquihydrate. The slope of the curve equals to $-\frac{Ea}{R}$. Figure S4: Projection along the a-axis of a periodic bond chain built from the interaction between prednisolone and water molecules. Hydrogen bonds involving water molecules are displayed in dashed green lines, and hydrogen bonds established directly between two prednisolone molecules are in dashed pink lines. Figure S5: Projection along the c-axis of a periodic bond chain built from the interaction between prednisolone and water molecules. Hydrogen bonds involving water molecules are displayed in dashed green lines, and hydrogen bonds established directly between two prednisolone molecules are in dashed pink lines. Figure S6: Projection along the a-axis of three periodic bond chains stacked along b and c. The water molecules ensured the cohesion between the PBC. One PBC is displayed in red. Figure S7: Projection along the c-axis of the periodic bond chains stacked along b and c. The water molecules ensured the cohesion between the PBC. One PBC is displayed in red. Figure S8: Projection along the a-axis of the crystal packing of prednisolone sesquihydrate. One PBC is displayed in red. Hydrogen bonds are in dashed lines depending on if a water molecule is involved (green) or not (pink). Figure S9: Projection along the c-axis of the crystal packing of prednisolone sesquihydrate. One PBC is displayed in red. Hydrogen bonds are in dashed lines depending on if a water molecule is involved (green) or not (pink). Figure S10: Superimposition of the two molecules of prednisolone present in the asymmetric unit of this sesquihydrate compound. Molecular modeling [6,7,8,9,10], explanation with Figure S11: Graphic obtained from the computed energy packing obtained by reference to the sesquihydrated packing. (Y scale in kcal·mol^−1^), and Table S1: Computed energy (in kcal·mol^−1^) of the sesquihydrated prednisolone and the different packing without a specific water molecule or pair of water molecules. Video S1: HT-microscopy of prednisolone sesquihydrate part A (100–111 °C). Video S2: HT-microscopy of prednisolone sesquihydrate part B (111–120 °C). Video S3: HT-microscopy of prednisolone sesquihydrate part C (120–150 °C).
