# Supplementary material for: Evidence of a New Crystalline Phase of Prednisolone Obtained from the Study of the Hydration–Dehydration Mechanisms of the Sesquihydrate
Source: Pharmaceutics. 2023 Jun 9;15(6):1694. doi: 10.3390/pharmaceutics15061694 (PMC10301155; doi:10.3390/pharmaceutics15061694)
Supplement: Supplementary file 1 [file pharmaceutics-15-01694-s001.zip › pharmaceutics-2409944-supplementary.pdf]

Hydration-Dehydration mechanisms of prednisolone sesquihydrate

Supplementary information

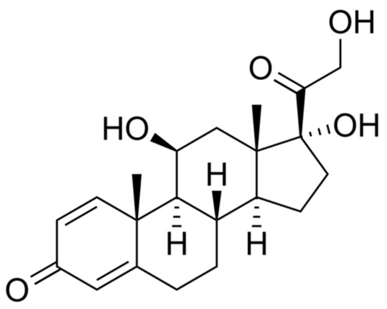

Figure S1: Molecular structure of Prednisolone.

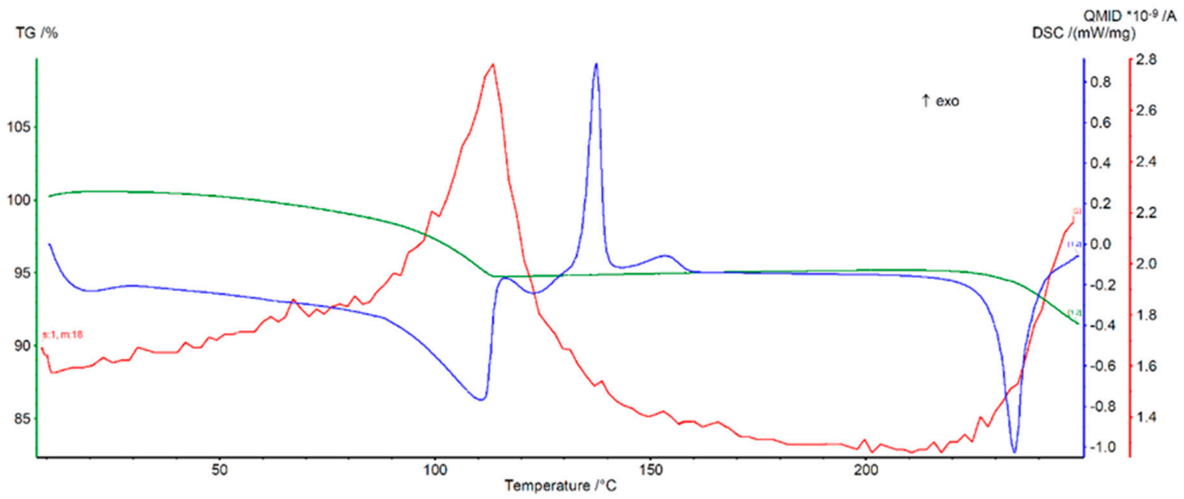

Figure S2: Concomitant Mass Spectrum variation for m/z= 18 (red curve) during TGA-DSC analysis of Prednisolone sesquihydrate (5K/min)

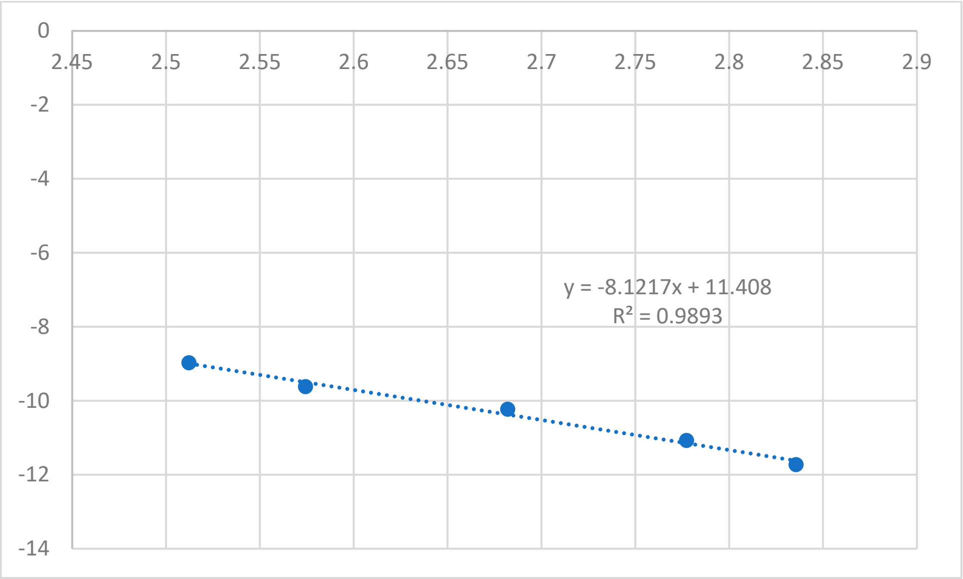

Figure S3: Kissinger plot curve: $\ln\left(\frac{\varphi}{T_p^2}\right) = f(\frac{1}{T_p})$ for dehydration peak of prednisolone sesquihydrate. Slope of the curve equals to $-\frac{Ea}{R}$

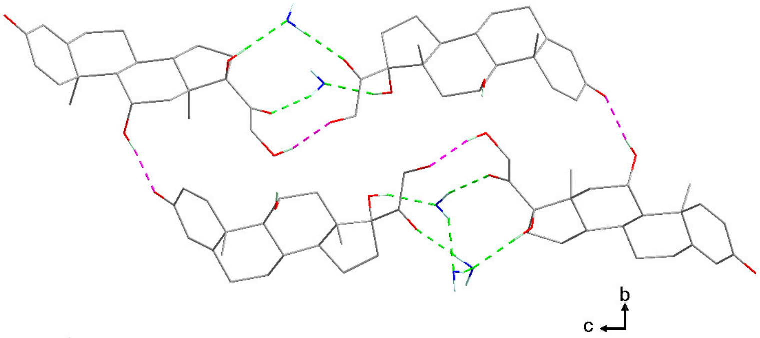

Figure S4: Projection along a axis of a periodic bond chain built from the interaction between prednisolone and water molecules. Hydrogen bonds involving water molecules are displayed in dashed green lines, and Hydrogen bonds established directly between two prednisolone molecules are in dashed pink lines.

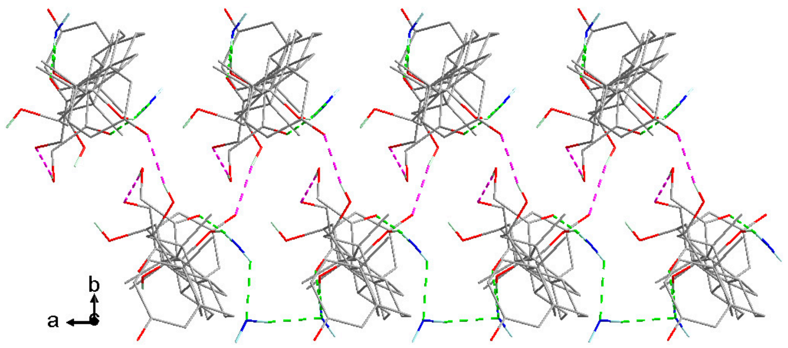

Figure S5: Projection along c axis of a periodic bond chain built from the interaction between prednisolone and water molecules. Hydrogen bonds involving water molecules are displayed in dashed green lines, and Hydrogen bonds established directly between two prednisolone molecules are in dashed pink lines.

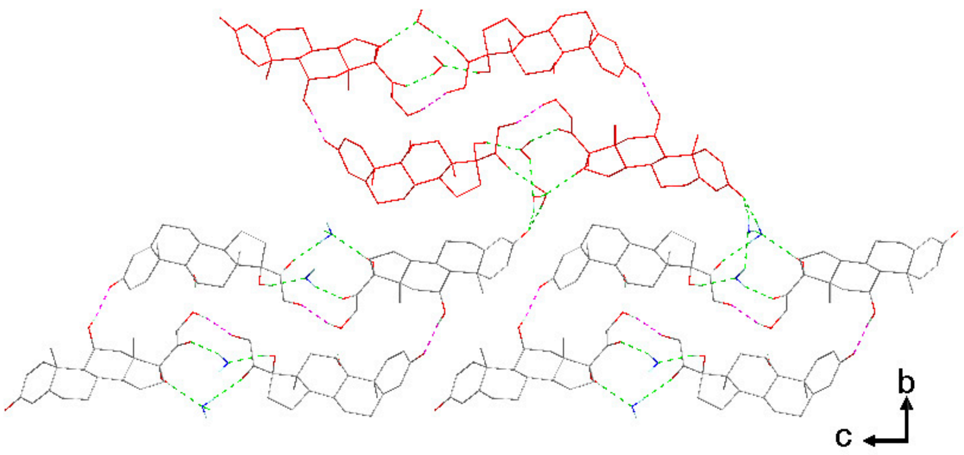

Figure S6: Projection along a axis of three periodic bond chains stacked along b and c. The water molecules ensured the cohesion between the PBC. One PBC is displayed in red.

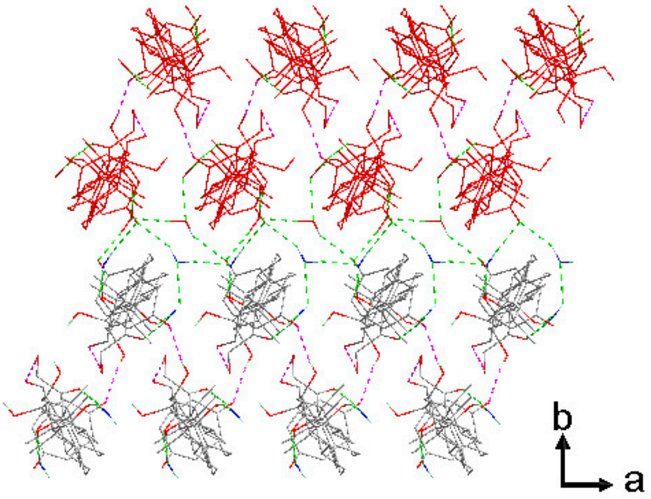

Figure S7: Projection along c axis of periodic bond chains stacked along b and c. The water molecules ensured the cohesion between the PBC. One PBC is displayed in red.

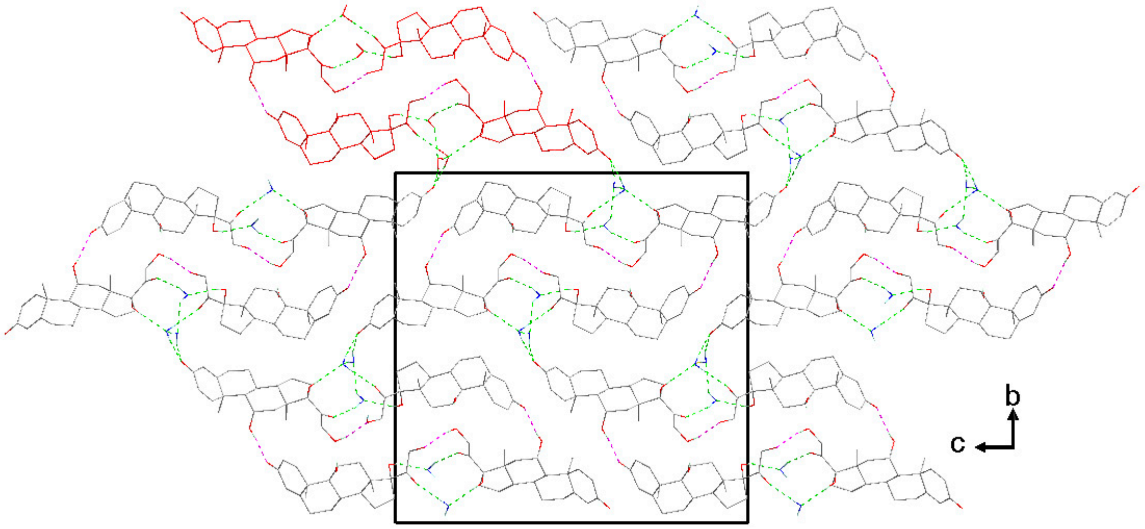

Figure S8: Projection along a axis of crystal packing of prednisolone sesquihydrate. One PBC is displayed in red. Hydrogen bonds are in dashed lines depending if a water molecule is involved (green) or not (pink).

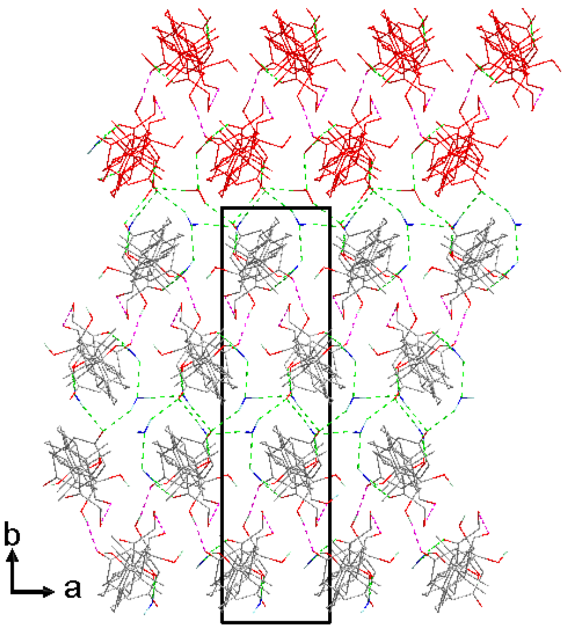

Figure S9: Projection along c axis of crystal packing of prednisolone sesquihydrate. One PBC is displayed in red. Hydrogen bonds are in dashed lines depending if a water molecule is involved (green) or not (pink).

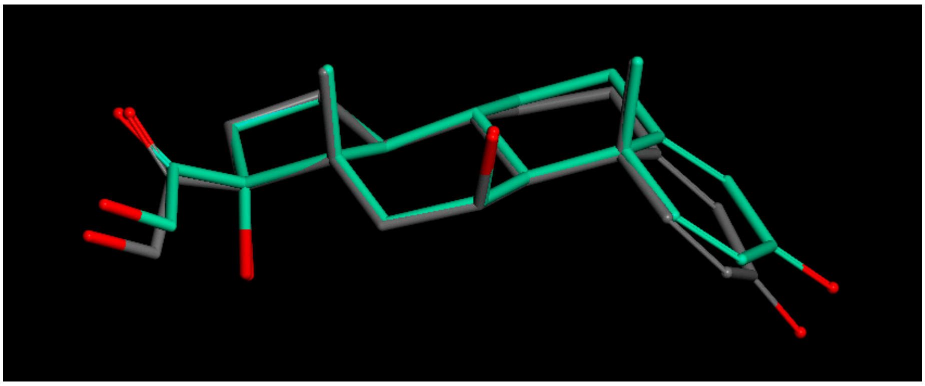

Figure S10: Superimposition of the two molecules of prednisolone present in the asymmetric unit of this sesquihydrate compound

Molecular Modeling

Molecular modeling calculations were performed using the Material Studio 2019 software[A]). Geometry optimizations were carried out with the forcite module using COMPASS II forcefield [B,C], and the smart algorithm. Charges were implemented using the forcefield, and non-bonding interactions were computed using the Ewald[D] option.

The method was based on the work of De Saint Jores et al.[E]. A unique cell was taken into account, geometry optimization was performed, and the energy of the packing was calculated (Eref).

Then, one water molecule at a time was removed, and the energy was computed for these three cases following the same procedure as for the E_{ref}, E_{woO11}, E_{woO12} and E_{woO13} were obtained, corresponding respectively to the packing without water molecule, O11, O12 and O13.

A second set of energy was computed, by removing two water molecule at the same time, energy was computed following the same procedure as describe above, and $E_{woO11O12}$, $E_{woO11O13}$, and $E_{woO12O13}$ were obtained, corresponding respectively to the packing without the couple of water molecules (O11, O12) , (O11, O13), and (O12, O13).

The easier a water molecule, or a pair of water can leave the packing, the lower the energetic loss has to be affecting the energy packing.

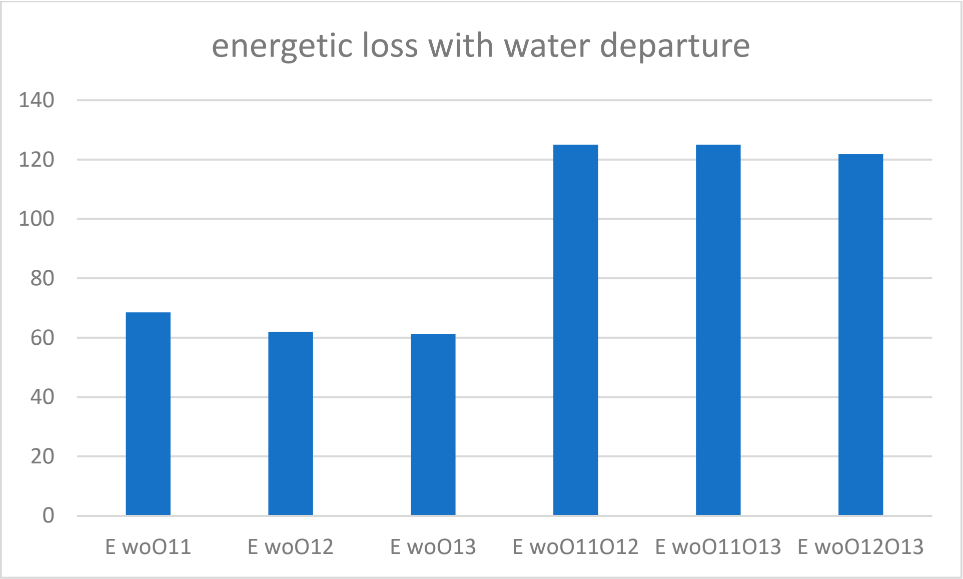

Figure S11: Graphic obtained from the computed energy packing obtained by reference to the sesquihydrated packing. (Y scale in $kcal.mol^{-1}$)

Table S1: Computed energy (in kcal.mol^{-1}) of the sesquihydrated prednisolone, and the different packing without a specific water molecule or pair of water molecules.

E ref (sesquihydrate prednisolone)	-294.8
Ewo O11	-226.3
Ewo O12	-232.8
Ewo O13	-233.5
EwoO11O12	-169.8
EwoO12O13	-173.0
EwoO11O13	-169.8

[A] BIOVIA, Material Studio, 2019 (19.1.0.2553), Dassault systems.

[B] :H. Sun, J. Phys. Chem. B 1998, 102 (38), 7338–7364. DOI: https://doi.org/10.1021/jp980939v;

[C] :H. Sun, P. Ren, J. R. Fried, Comput. Theor. Polym. Sci. 1998, 8 (1), 229–246. DOI: https://doi.org/10.1016/S1089-3156(98)00042-7)

[D] P. P. Ewald, Ann. Phys. 1921, 369 (3), 253–287. DOI: https://doi.org/10.1002/andp.19213690304

[E] *Limitations of preferential enrichment: a case study on tryptophan ethyl ester hydrochloride, Chem. Eng. Technol, 2019, 42, n°7, 1500-1504, DOI:10.1002/ceat.201800702*
